# The Risk of Functional Limitations After Driving Cessation Among Older Japanese Adults: The JAGES Cohort Study

**DOI:** 10.2188/jea.JE20180260

**Published:** 2020-08-05

**Authors:** Hiroshi Hirai, Masao Ichikawa, Naoki Kondo, Katsunori Kondo

**Affiliations:** 1 Department of Regional Social Management, Faculty of Life and Environmental Sciences, University of Yamanashi, Yamanashi, Japan; 2 Department of Global Public Health, Faculty of Medicine, University of Tsukuba, Ibaraki, Japan; 3 Department of Health Education and Health Sociology, School of Public Health, The University of Tokyo, Tokyo, Japan; 4 Department of Social Preventive Medical Sciences, Center for Preventive Medical Sciences, Chiba University, Chiba, Japan; 5 Department of Gerontological Evaluation, Center for Gerontology and Social Science, National Center for Geriatrics and Gerontology, Aichi, Japan; 6 Center for Well-Being and Society, Nihon Fukushi University, Aichi, Japan

**Keywords:** ageing, automobile driving, transportation, activities of daily living

## Abstract

**Background:**

Population ageing and stringent licensing policies will increase the number of older drivers who stop driving. Adverse health outcomes owing to driving cessation and their prevention are emerging concerns. Therefore, we longitudinally examined the impact of driving cessation and alternative transportation use after cessation on the risk of functional limitations in a cohort of community-dwelling people (65 years and older) in Japan.

**Methods:**

Using cohort data of those who drove as of 2006/07, we compared the risk of functional limitations between 2,704 current drivers and 140 former drivers (who stopped driving by 2010). Of the former drivers, 77 did not use public transportation or bicycles after driving cessation (thus losing independent mobility). We calculated the hazard ratios (HRs) for the incidence of functional limitations with 95% confidence intervals (CIs) based on the Cox proportional hazards model with the covariates influencing the functional limitations.

**Results:**

From 2010 through 2016, 645 people had functional limitations, which included 38, 82, and 119 per 1,000 person-years among current drivers, former drivers who used public transportation or bicycles, and former drivers who were only driven by others, respectively (HR 1.69; 95% CI, 1.15–2.49 and HR 2.16; 95% CI, 1.51–3.10, relative to current drivers).

**Conclusion:**

Driving cessation is associated with an increased risk of functional limitations among older adults, but this risk might be alleviated if they are able to maintain independent mobility using public transportation or bicycles after driving cessation.

## INTRODUCTION

In Japan, the number of older drivers is increasing in the driving population. Consequently, motor-vehicle collisions (MVCs) caused by older drivers are also on the rise.^1^ In 2015, 17 million people aged 65 years or older were licensed to drive (21% of the driving population), and the percentage of at-fault MVCs by them nearly doubled in the past decade, from 12% in 2006 to 20% in 2015.^2^^,^^3^ While older drivers have fewer at-fault MVCs per kilometre travelled than younger drivers, their rate gradually increases with age.^4^ This trend in MVCs among older drivers has impelled the National Police Agency, which oversees the licensing of drivers, to impose mandatory driving lessons, including on-road driving assessments and cognitive screenings of drivers from age 70 and 75 years, respectively, at the time of licence renewal, and to encourage older drivers to surrender their driving licences voluntarily.^5^ From March 2017, drivers who are suspected to have dementia during cognitive screenings are required to receive a formal diagnosis, and if they actually have dementia, their driving licence will be revoked or suspended.^5^

Older drivers never cause MVCs once they stop driving; however, driving cessation restricts their mobility and affects their well-being. A recent systematic review of studies of the impact of driving cessation identified the following adverse consequences: depressive symptoms; significant declines in general health, physical functioning, cognitive functioning, and social engagement; and greater risks of admission to long-term care facilities and mortality.^6^ A study conducted in Japan suggested that driving cessation increases the risk of functional limitations.^7^ Moreover, a study from Denmark reported that the introduction of cognitive screening of older drivers induced a shift from driving to using unprotected modes of transportation, such as walking and cycling, resulting in an increase in fatal injuries among older road users.^8^ Therefore, driving cessation should not be encouraged only for the sake of traffic safety without considering the adverse social and health outcomes that could be prevented by continuing driving. Meanwhile, alternative transportation together with walkable/cyclable roads should be made available for an increasing number of older adults who will retire from driving in the near future.

Japan’s licensing policies against older drivers are becoming stringent, as mentioned above, and older drivers might be prompted to stop driving. These policies affect older adults’ well-being if they are actually able to prevent the adverse outcomes by continuing driving. On the other hand, it would be useful to examine whether alternative transportation use after driving cessation buffers the adverse outcomes. A recent study based on a nationally representative cohort in the United States reported that frail older adults were more likely to become non-drivers than the non-frail.^9^ Therefore, in the present study, we longitudinally investigated the impact of driving cessation and alternative transportation use after cessation on the risk of functional limitations among frail and non-frail older adults, using cohort data from the Japan Gerontological Evaluation Study (JAGES) of older drivers who either continued or stopped driving over a 6-year period.

## METHODS

### Data

The JAGES is a cohort study of community-dwelling people aged 65 years or older who have no physical or cognitive disabilities and who were not receiving long-term care insurance (LTCI) benefits at the time of the baseline survey. The JAGES, launched in 1999 as the Aichi Gerontological Evaluation Survey in two municipalities in Aichi Prefecture, is a prospective and retrospective investigation of the course of life of individual older adults. The project has been extended to 30 municipalities in 14 prefectures and it involved 138,000 participants as of 2013. The JAGES protocol was approved by the Ethics Committee on the Research of Human Subjects at Nihon Fukushi University (no. 10-05). The details have been given separately.^10^

**Figure 1.**
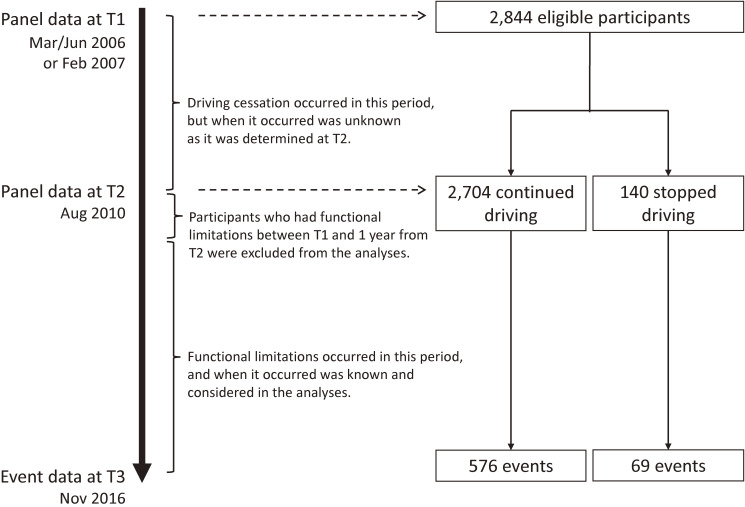
Flow chart of data collection and participants

The data for the JAGES were collected mostly through self-administered questionnaires that were mailed to a random sample of functionally independent individuals, aged 65 years or older, living in the participating municipalities. In the present study, we used the data from four municipalities in Aichi Prefecture because the outcome information of interest was readily available only in these municipalities. The panel data that we used were derived from a survey conducted in March 2006, June 2006, or February 2007 (the time varied between the municipalities), and again in August 2010 (hereinafter, referred to as T1 and T2, respectively), in which the respondents were asked about their modes of transportation. The outcome variable, functional limitations as described below, was assessed in November 2016 (T3) (Figure 1).

Of the 8,349 individuals who responded at T1 and T2, 4,925 reported that they drove cars at T1, and were participants in the study. However, 270 of them were excluded from the analyses because their frequency of travelling outside their homes by any means of transportation was reportedly less than once a week at T1, indicating that they were home-bound and, thus, inactive drivers. An additional 1,717 were excluded because of missing information for the variables studied in the analyses. Furthermore, we excluded 94 who were certified for LTCI benefits within a year from T2 as well as between T1 and T2, assuming that they had unmeasured preconditions to have functional limitations irrespective of driving cessation. Therefore, the sample size of the present study was 2,844 (Figure 1).

### Measures

The certification for LTCI benefits, which is an indicator of functional limitations, was the study’s outcome variable. This is a public insurance system that provides long-term care for those aged 65 years or older if they require long-term care owing to their physical or mental conditions. To receive these benefits, one has to receive approval through standard certification procedures.^11^ The JAGES collected information about the certification for LTCI benefits or deaths from the municipalities, which were also the LTCI insurers. We considered the date of certification for LTCI benefits or death as the onset of functional limitations, while those who moved out of the municipalities (31 out of 2,844) were censored on the date of its notification at the municipal offices.

The data for the exposure variable and covariates were obtained mostly through self-administered questionnaires. The exposure variable was driving cessation. We assumed that the participants had stopped driving if they did not report that they drove cars at T2; they are hereinafter referred to as former drivers. Among the former drivers, we determined whether they used public transportation or bicycles at T2 or if they were only driven by others. Former drivers who used public transportation or bicycles as well as users of both were combined because they were considered to use an independent mode of transportation, which is different in its nature from a passive mode (ie, driven by others). Those who continued driving are referred to as current drivers. Their utilization of public transportation or bicycles at T2 was also determined.

The covariates at T2 (baseline characteristics) included the participants’ age; sex; education; body mass index (BMI); self-rated health; self-reported diseases under treatment, including arthritis, osteoporosis, and fractures; depressive symptoms; physical and cognitive function; frequency of travelling outside of the home; ability to travel outside of the home alone on a bus or a train; shopping habits for daily necessities; visits to friends’ homes; and requests for advice from others. These variables, which are associated with the risk of functional limitations, were selected based on previous findings of studies conducted in Japan.^7^^,^^12^

Participants with a BMI <18.5 kg/m^2^ were classified as underweight, and those with a BMI ≥25.0 kg/m^2^ as overweight. Self-rated health was measured on a 4-point scale ranging from poor to excellent. Depressive symptoms were measured using the Geriatric Depression Scale, which consists of 15 items requiring yes/no responses and has a total possible score ranging from 1 to 15, representing the number of depressive symptoms.^13^^,^^14^ In this study, the cut-off scores for levels of severity were 0–4 (not depressed), 5–9 (mildly depressed), and 10–15 (depressed).

Physical and cognitive function was assessed using the Kihon Checklist, which is a screening tool in the aforementioned LTCI procedure.^15^ This checklist consists of 25 items in seven domains (activities of daily living, physical strength, nutrition, oral function, isolation, memory, and mood) requiring yes/no responses. The checklist score therefore ranges from 0 to 25. To determine reduced physical and cognitive function among the participants, we adopted a cut-off point used in the LTCI procedure: 3 of 5 physical strength items for physical function and 1 of 3 memory items for cognitive function.

An additional covariate was population density of a school district where each participant lived. This was calculated using small area census data and land information obtainable through jSTAT MAP of the Statistics Bureau and the National Land Numerical Information of the Ministry of Land, Infrastructure, Transport and Tourism, respectively. We included this variable as a surrogate indicator of access to public transportation given the correlation between population density and public transportation use.^16^ We also included public transportation or bicycle use as a covariate.

To identify frail adults among the participants, we used the Kihon Checklist as well. The checklist score of 8 or greater indicates a frail status and a score of 4 to 7 indicates a pre-frail status. The checklist score has been validated to estimate the frailty status as defined by the Cardiovascular Health Study frailty index.^17^^,^^18^

### Analysis

First, we compared the participants’ baseline characteristics by their driving status at T2 (between current and former drivers), with reference to the effect size of eta squared for continuous variables and Cramer’s V for categorical variables. In this group comparison, an effect size of 0.01 and 0.07 indicates a small difference for continuous and categorical variables, respectively; corresponding effect sizes of 0.06 and 0.21 indicate moderate differences.^19^ This comparison was made among all participants and specifically among frail participants for a stratified analysis, which is explained later.

Next, we calculated the rate of functional limitations per 1,000 person-years over the follow-up period from August 2010 (T2) to November 2016 (T3) among all current and former drivers and among frail current and former drivers. Then, we estimated the impact of driving cessation and alternative transportation use on the subsequent functional limitations using the Cox proportional hazards model with the aforementioned covariates. We obtained both crude and adjusted hazard ratios (HRs) with their 95% confidence intervals (CIs) for the incidence of functional limitations. In this model, we examined whether public transportation or bicycle use alleviated the impact of driving cessation by entering an interaction term of these variables. Further, we conducted a stratified analysis by frailty status to examine the impact of driving cessation particularly among the frail participants to suggest policy implications given their higher chance of stopping driving.^9^ The stratified analysis among the non-frail and pre-frail participants was not practical owing to the small number of former drivers and the outcomes.

Finally, we divided the participants into three groups (current drivers, former drivers who used public transportation or bicycles, and former drivers who were only driven by others) and compared the risk of functional limitations across the three groups using the same model. This was also intended to see whether public transportation or bicycle use alleviated the impact of driving cessation.

## RESULTS

The participants’ baseline characteristics were compared between current and former drivers among all participants (Table 1) and among the frail participants (Table 2). Generally, former drivers had lower health status and were less active than current drivers in both sets of participants. According to the effect size, the group difference was more prominent with regard to their daily activities than their health status. Regarding population density, a surrogate indicator of access to public transportation, there was no clear group difference, but public transportation or bicycle use was less among current drivers than former drivers.

**Table 1.** The characteristics of current and former drivers, as of August 2010

	Current drivers (*n* = 2,704)		Former drivers (*n* = 140)		Effect size^a^
	
	*n*	%	*n*	%	
Age, years, mean (standard deviation)	70.1	4.3	73.6	5.8	0.028
Males	1,782	65.9%	74	52.9%	0.059
Education					
≥13 years	419	15.5%	18	12.9%	0.045
10–12 years	931	34.4%	46	32.9%	
6–9 years	1,325	49.0%	73	52.1%	
<6 years	21	0.8%	1	0.7%	
Unknown	8	0.3%	2	1.4%	
Body mass index					
Normal	1,966	72.7%	89	63.6%	0.073
Underweight (BMI <18.5 kg/m^2^)	143	5.3%	18	12.9%	
Overweight (BMI ≥27.5 kg/m^2^)	595	22.0%	33	23.6%	
Self-rated health, poor^b^	50	1.8%	3	2.1%	0.075
Self-reported diseases under treatment, yes	356	13.2%	22	15.7%	0.016
Physical function, low^c^	433	16.0%	50	35.7%	0.114
Cognitive function, low^c^	865	32.0%	47	33.6%	0.007
Depressive symptoms (GDS scores)^d^					
Non-depressed (0–4)	2,115	78.2%	91	65.0%	0.086
Mildly depressed (5–9)	467	17.3%	32	22.9%	
Depressed (10–15)	122	4.5%	17	12.1%	
Frequency of going outside of the home					
Almost everyday	1,796	66.4%	56	40.0%	0.203
2–3 days a week	712	26.3%	43	30.7%	
1 day a week	137	5.1%	20	14.3%	
Once or twice a month	35	1.3%	15	10.7%	
Several times a year	20	0.7%	5	3.6%	
Never	4	0.1%	1	0.7%	
Going outside of the home alone, no	118	4.4%	23	16.4%	0.120
Shopping for daily necessities, no	21	0.8%	7	5.0%	0.093
Visiting friends’ homes, no	580	21.4%	57	40.7%	0.100
Being asked for advice, no	316	11.7%	35	25.0%	0.088
Population density					
≥2,000/km^2^	881	32.6%	49	35.0%	0.013
1,000–1,999/km^2^	447	16.5%	21	15.0%	
<1,000/km^2^	1,376	50.9%	70	50.0%	
Public transportation or bicycle use, no	1,914	70.8%	77	55.0%	0.075

BMI, body mass index; GDS, Geriatric Depression Scale.

^a^Eta-squared for age and Cramer’s V for other variables.

^b^Self-rated health was measured on a 4-point scale ranging from 1 (poor) to 4 (excellent).

^c^Physical and cognitive function was assessed based on the Kihon Checklist.

^d^Depressive symptoms were measured using the Geriatric Depression Scale with a total possible score of 0–15.

**Table 2.** The characteristics of frail current and former drivers, as of August 2010

	Frail current drivers (*n* = 1,464)		Frail former drivers (*n* = 105)		Effect size^a^
	
	*n*	%	*n*	%	
Age, years, mean (standard deviation)	70.7	4.5	74.1	5.9	0.033
Males	953	65.1%	56	53.3%	0.061
Education					
≥13 years	196	13.4%	14	13.3%	0.088
10–12 years	475	32.4%	33	31.4%	
6–9 years	774	52.9%	55	52.4%	
<6 years	17	1.2%	1	1.0%	
Unknown	2	0.1%	2	1.9%	
Body mass index					
Normal	1,042	71.2%	62	59.0%	0.090
Underweight (BMI <18.5 kg/m^2^)	105	7.2%	17	16.2%	
Overweight (BMI ≥27.5 kg/m^2^)	317	21.7%	26	24.8%	
Self-rated health, poor^b^	45	3.1%	3	2.9%	0.063
Self-reported diseases under treatment, yes	258	17.6%	21	20.0%	0.016
Physical function, low^c^	424	29.0%	50	47.6%	0.102
Cognitive function, low^c^	694	47.4%	41	39.0%	0.042
Depressive symptoms (GDS scores)^d^					
Non-depressed (0–4)	941	64.3%	56	53.3%	0.078
Mildly depressed (5–9)	405	27.7%	32	30.5%	
Depressed (10–15)	118	8.1%	17	16.2%	
Frequency of going outside of the home					
Almost everyday	876	59.8%	41	39.0%	0.224
2–3 days a week	444	30.3%	27	25.7%	
1 day a week	96	6.6%	17	16.2%	
Once or twice a month	29	2.0%	14	13.3%	
Several times a year	15	1.0%	5	4.8%	
Never	4	0.3%	1	1.0%	
Going outside of the home alone, no	104	7.1%	23	21.9%	0.136
Shopping for daily necessities, no	19	1.3%	7	6.7%	0.105
Visiting friends’ homes, no	455	31.1%	52	49.5%	0.099
Being asked for advice, no	258	17.6%	32	30.5%	0.083
Population density					
≥2,000/km^2^	484	33.1%	39	37.1%	0.026
1,000–1,999/km^2^	240	16.4%	14	13.3%	
<1,000/km^2^	740	50.5%	52	49.5%	
Public transportation or bicycle use, no	1,089	74.4%	60	57.1%	0.097

BMI, body mass index; GDS, Geriatric Depression Scale.

^a^Eta-squared for age and Cramer’s V for other variables.

^b^Self-rated health was measured on a 4-point scale ranging from 1 (poor) to 4 (excellent).

^c^Physical and cognitive function was assessed based on the Kihon Checklist.

^d^Depressive symptoms were measured using the Geriatric Depression Scale with a total possible score of 0–15.

Table 3 shows the number and rate of functional limitations among all participants and among the frail participants, together with the HR for the incidence of functional limitations that was estimated using the Cox proportional hazards model. Among all participants, 645 had functional limitations by T3, as verified by their certification for LTCI benefits during the follow-up period, which included 576 current drivers (38 per 1,000 person-years) and 69 former drivers (101 per 1,000 person-years). Former drivers had a higher rate of functional limitations than current drivers (HR 2.09; 95% CI, 1.35–3.24). The results appeared to be similar for all participants and for the frail participants.

**Table 3.** The risk of functional limitations among current drivers and former drivers (who used public transportation or bicycles, and who were only driven by others) over a 6-year period from August 2010 to November 2016

	*N*	*n*	rate	Hazard ratio (95% confidence interval)	

				Unadjusted	Adjusted^a^
Model 1					
All participants					
Current drivers	2,704	576	37.6	Reference	Reference
Former drivers	140	69	101.1	2.73 (1.81–4.12)	2.09 (1.35–3.24)
Frail participants^b^					
Current drivers	1,464	404	50.3	Reference	Reference
Former drivers	105	58	119.7	2.38 (1.48–3.84)	2.00 (1.20–3.32)

Model 2					
All participants					
Current drivers	2,704	576	37.6	Reference	Reference
Former drivers who used public transportation or bicycles	63	27	82.3	2.31 (1.62–3.30)	1.69 (1.15–2.49)
Former drivers who were only driven by others	77	42	118.6	3.51 (2.52–4.90)	2.16 (1.51–3.10)
Frail participants^b^					
Current drivers	1,464	404	50.3	Reference	Reference
Former drivers who used public transportation or bicycles	45	21	92.6	2.03 (1.35–3.03)	1.60 (1.02–2.50)
Former drivers who were only driven by others	60	37	143.5	3.10 (2.17–4.42)	2.37 (1.61–3.50)

*Note*: *N* indicates the number of the participants in each category, *n* indicates the number of the participants who had functional limitations during the follow-up, and the rate is the number of functional limitations per 1,000 person-years.

^a^Adjusted in the Cox proportional hazards model (1 and 2) for participants’ age, sex, education, body mass index, self-rated health, self-reported diseases under treatment, depressive symptoms, physical and cognitive function, frequency of travelling outside of the home, ability to travel outside of the home alone on a bus or a train, shopping habits for daily necessities, visits to friends’ homes, requests for advice from others, and population density. In addition, public transportation or bicycle use was adjusted in the model 1.

^b^Those with a Kihon Checklist score >7.

Public transportation or bicycle use was associated with a lower risk of functional limitations (HR 0.82; 95% CI, 0.68–1.00 among all participants and HR 0.77; 95% CI, 0.60–0.98 among the frail participants) but it did not clearly alleviate the impact of driving cessation as the interaction term of these variables was not significant with a *P*-value of 0.77 and 0.78, respectively (not shown in Table 3). However, a comparison across current drivers, former drivers who used public transportation or bicycles, and former drivers who were only driven by others showed a clear gradient in the rate of functional limitations (38, 82, and 119 per 1,000 person-years, respectively), with a higher rate among former drivers who were only driven by others (HR 2.16; 95% CI, 1.51–3.10), followed by former drivers who used public transportation or bicycles (HR 1.69; 95% CI, 1.15–2.49) as compared with current drivers. Again, the results appeared to be similar for all participants and for the frail participants.

## DISCUSSION

Our findings suggest that the risk of functional limitations might increase among older adults once they stop driving, while this risk might be somewhat alleviated if they are able to maintain independent mobility by using public transportation or bicycles. The same can be said for frail older adults. This implication is supported by previous findings, which were based on the same cohort as in the present study, that if frail adults engage in active living, such as going out every day and meeting friends, they are more likely to be able to improve their frailty status.^20^ Indeed, independent mobility helps with active living and enables us to meet our daily need to travel for shopping, social activities, leisure, and visiting friends, all of which influence our well-being.^21^

Older adults can maintain independent mobility by driving or using public transportation or bicycles; as such, they should be assisted with driving safely or using these transportation alternatives conveniently and safely, irrespective of their frailty status. However, Japan’s licensing policies against older drivers have been increasingly toughened and they are more stringent than in many other industrialised countries.^22^^–^^24^ Therefore, it is worth investigating whether such policies prompt premature driving cessation among older adults and whether their MVCs are actually reduced by these policies. So far, a mandatory driving lesson for drivers aged 70 years or older at licence renewal has not been shown to be effective for reducing their at-fault MVCs.^1^ In addition, the public transport network has been shrinking, especially outside the major cities in Japan. In fact, the number of passengers carried by public bus and regional railways has decreased over the past 25 years by 35% and 20%, respectively.^25^ Meanwhile, the governmental agencies foresee an increasing number of older adults who will stop driving, and the development of a mobility support system for older adults is underway.^26^

Our challenge in causal inference was to take into account the trajectory of driving cessation and its impact. With the following analyses, we improved our causal inference. First, we excluded the participants who were certified for LTCI benefits within a year from T2 as well as between T1 and T2, assuming that they had unmeasured preconditions for functional limitations. This helped minimize the possibility of reverse causation. Second, we performed stratified analyses restricting to frail participants, and this helped improve our causal inference because current drivers and former drivers became comparable at T2 in terms of frailty. In this way, we elucidated the impact of driving cessation and alternative transportation use on the risk of functional limitations, though unmeasured confounding effects still remained uncontrolled.

In conclusion, driving cessation is associated with an increased risk of functional limitations among older adults, but this risk might be alleviated if they are able to maintain independent mobility by using public transportation or bicycles after driving cessation.
